# Supplementary material for: Overcoming therapeutic resistance in oncolytic herpes virotherapy by targeting IGF2BP3-induced NETosis in malignant glioma
Source: Nat Commun. 2024 Jan 2;15:131. doi: 10.1038/s41467-023-44576-2 (PMC10762148; doi:10.1038/s41467-023-44576-2)
Supplement: Supplementary file 1 — Supplementary Information [file 41467_2023_44576_MOESM1_ESM.pdf]

Overcoming Therapeutic Resistance in Oncolytic Herpes Virotherapy by Targeting IGF2BP3-Induced NETosis in malignant glioma

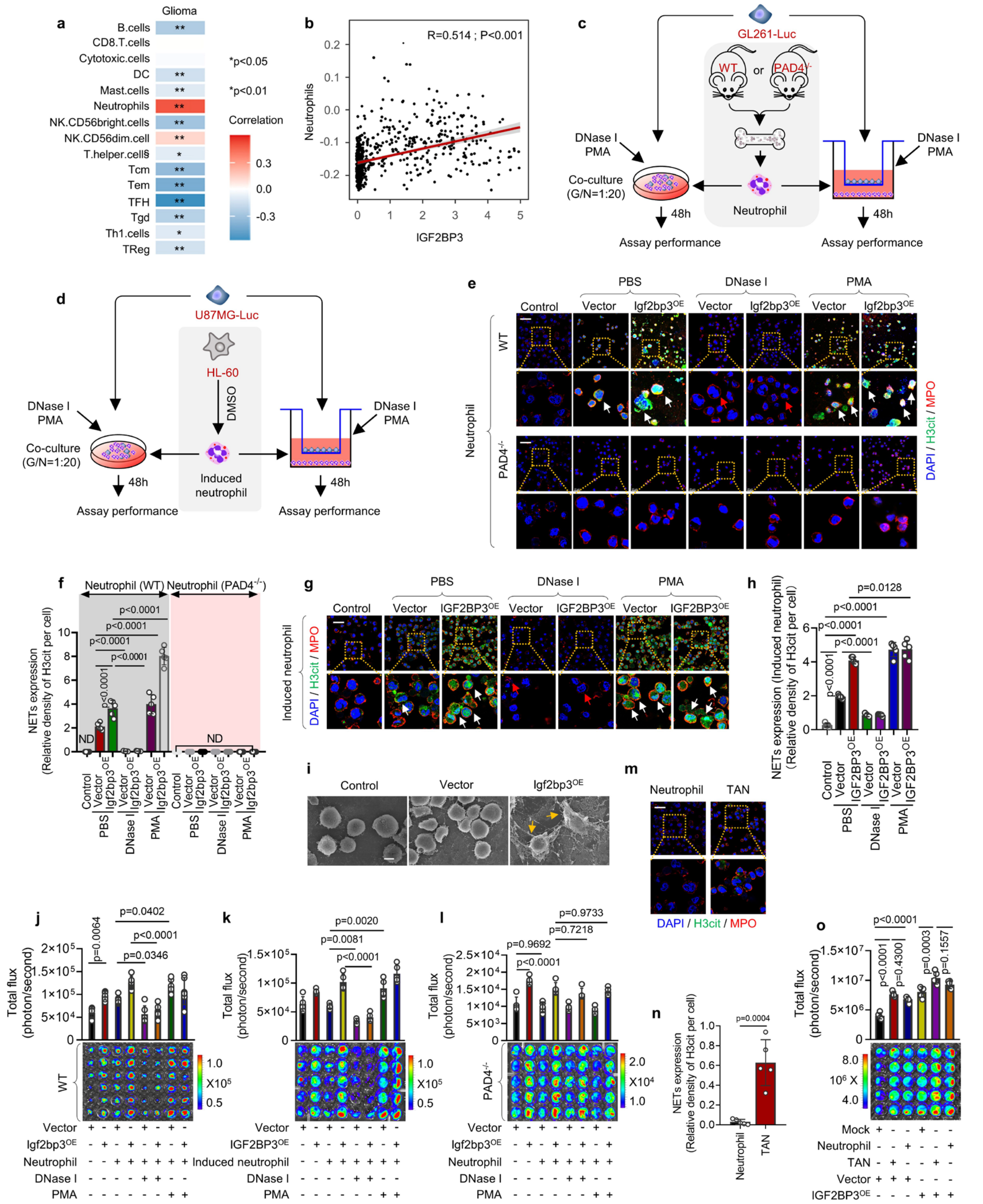

Supplementary Figure 1. IGF2BP3 promotes NETs formation and glioma survival

(a, b) IGF2BP3 mRNA levels were correlated with ssGSEA scores of tumor-infiltrating immune cells in glioma samples (n= 596) using Spearman's test. Negative correlations were indicated in blue, and positive correlations were denoted in red**(a)**. Spearman's test identified positive correlations between IGF2BP3 mRNA levels and neutrophil infiltration abundance via ssGSEA in glioma samples (n= 596) **(b)**.

(c, d) The schematic depicts the contacted co-culture assay and uncontacted transwell

assay. In panel **(c)**, bone marrow neutrophils from wild-type (WT) or PAD4 knockout ($PAD4^{-/-}$) mice were isolated. In panel **(d)**, "U87MG-Luc" refers to luciferase-expressing U87MG cells. Induced neutrophils were derived from DMSO-pretreated HL-60 cells, as previously described[1]. "G/N=1:20" signifies the glioma cell to neutrophil ratio.

(e-h) Representative immunofluorescence images **(e, g)** and quantification **(f, h)** of NET formation in GL261 cell/bone marrow neutrophil and U87MG cell/induced neutrophil (HL-60) uncontacted transwell assays. "Control" denotes unstimulated neutrophils in vitro. White arrows indicate NETs co-stained with H3cit, MPO, and DAPI, while red arrows denote intact neutrophils. Scale bars, 20 μm. "ND" signifies undetected. Representative images of n = 5 biological independent samples.

(i) Electron microscopic analysis of bone marrow neutrophils (wild-type) co-cultured with GL261-Luc cells. "Control" indicates neutrophils unstimulated with GL261 glioma cells in vitro. Yellow arrows indicate NETs. Scale bars, 5 μm. Representative images of n = 3.

(j-l) NETs-induced GL261 cell survival in the contacted co-culture assay was assessed using a luciferase assay. n = 6 for panel **(j)** and n = 5 for panels **(k)** and **(l)**, representing biological independent samples.

(m, n) Representative immunofluorescence images **(m)** and quantification **(n)** of NET formation in control bone marrow neutrophil and TAN unstimulated with glioma cells in vitro. Scale bars,20 μm. Representative images of n = 5. n = 5 biological independent samples.

(o) The effect of TAN and control neutrophils on GL261 cell survival in the contacted co-culture assay. n = 5 biological independent samples.

Statistical significance was determined using one-way ANOVA in **(f), (h), (j-l), (o)** or two-tailed Student's t-test in **(n).** Data represent the mean ± SD. Source data are provided in the Source Data file.

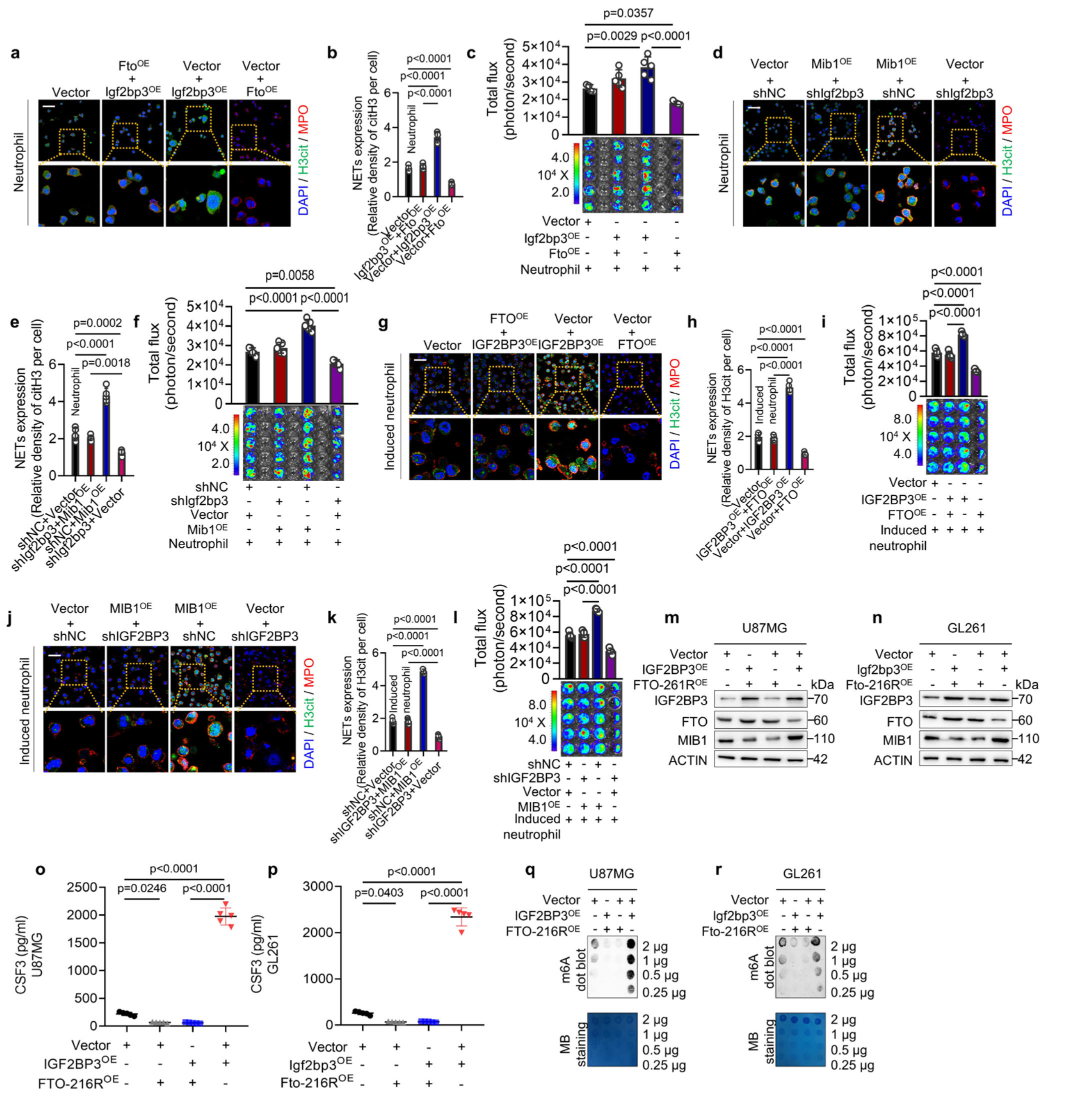

Supplementary Figure 2. Disrupting the IGF2BP3/MIB1/FTO feedback loop abolishes NETosis

(a, b, d, e) Immunofluorescence images **(a, d)** and quantification **(b, e)** of NET formation in the GL261 cell/bone marrow neutrophil uncontacted transwell assay with the indicated treatments. Scale bars: 20 μm. Representative images of n = 5. n = 5 biological independent samples.

(c, f) The NET-induced survival of GL261 cells in contacted co-culture assay with the indicated treatments was detected using a luciferase assay. n = 5 biological independent samples.

(g, h, j, k) Immunofluorescence images **(g, j)** and quantification **(h, k)** of NET formation in the U87MG cell/induced neutrophil (HL-60) uncontacted transwell assay with the indicated treatments. Scale bars, 20 μm. Representative images of n = 5. n = 5 biological independent samples.

(i, l) The NET-induced survival of U87MG cells in contacted co-culture assay with the indicated treatments was detected using a luciferase assay. n = 5 biological independent samples.

(m, n) Rescue experiments showed the effect of FTO-216R mutant transfection on

IGF2BP3/MIB1/FTO pathway. n = 3 biological independent experiments.

(o, p) ELISA assay was used to detect the effect of FTO-216R mutant on the protein level of CSF3. n = 5 biological independent samples.

(q, r) The effects of FTO-216R mutant transfection on IGF2BP3-regulated overall m6A levels.

Statistical significance was determined using one-way ANOVA in **(b), (c), (e), (f), (h), (i), (k), (l), (o), (p).** Data represent the mean ± SD. Source data are provided in the Source Data file.

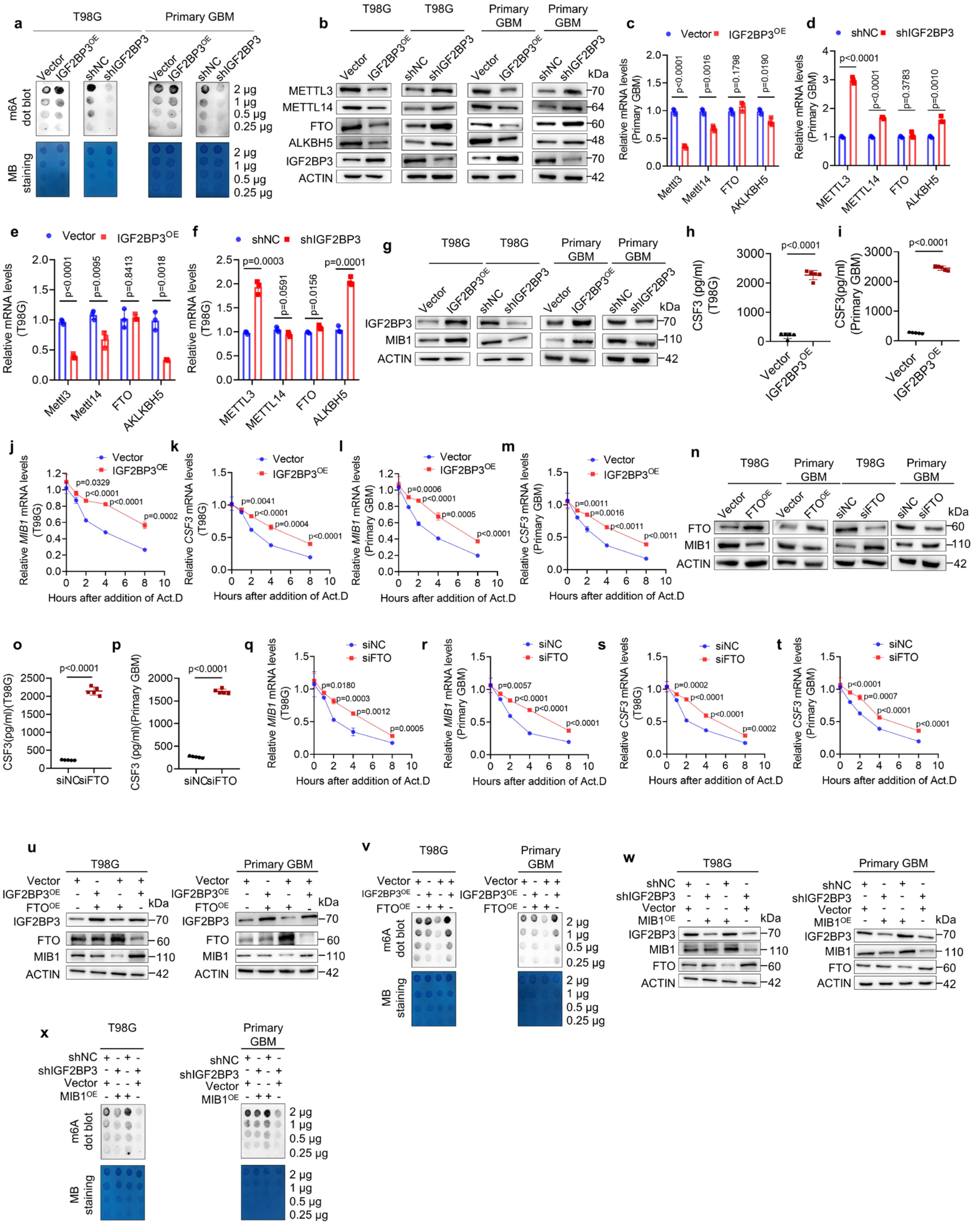

Supplementary Figure 3. IGF2BP3/MIB1/FTO loop is confirmed in multiple glioma

cell models

(a) The impact of IGF2BP3 on total m6A levels. Methylene blue (MB) staining served as the loading control.

(b-f) The protein(b) and mRNA **(c, d, e, f)** changes in m6A-related enzymes in T98G and primary human GBM cells with specific treatments. n =3 biological independent experiments.

(g) The impact of IGF2BP3 on the MIB1 protein expression in T98G and primary human GBM cells. n = 3 biological independent experiments.

(h, i) The effect of IGF2BP3 on the protein levels of CSF3 detected by ELISA assay. n = 5 biological independent samples.

(j-m) The effect of IGF2BP3 on the MIB1 and CSF3 mRNA stability. n = 3 biological independent experiments.

(n) The impact of FTO on the MIB1 protein expression in T98G and primary human GBM cells. n = 3 biological independent experiments.

(o, p) The effect of FTO knockdown on the protein levels of CSF3 detected by ELISA assay. n = 5 biological independent samples.

(q-t) The effect of FTO on the MIB1 and CSF3 mRNA stability. n = 3 biological independent experiments.

(u, w) Rescue experiments showed the effects of FTO **(u)** or MIB1 **(w)** interruption on IGF2BP3/MIB1/FTO pathway. n = 3 biological independent experiments.

(v, x) The effects of FTO **(v)** or MIB1 **(x)** interruption on IGF2BP3-regulated overall m6A levels.

Statistical significance was determined using two-tailed Student's t-test in **(c-f), (h-m), (o-t).** Data represent the mean ± SD. Source data are provided in the Source Data file.

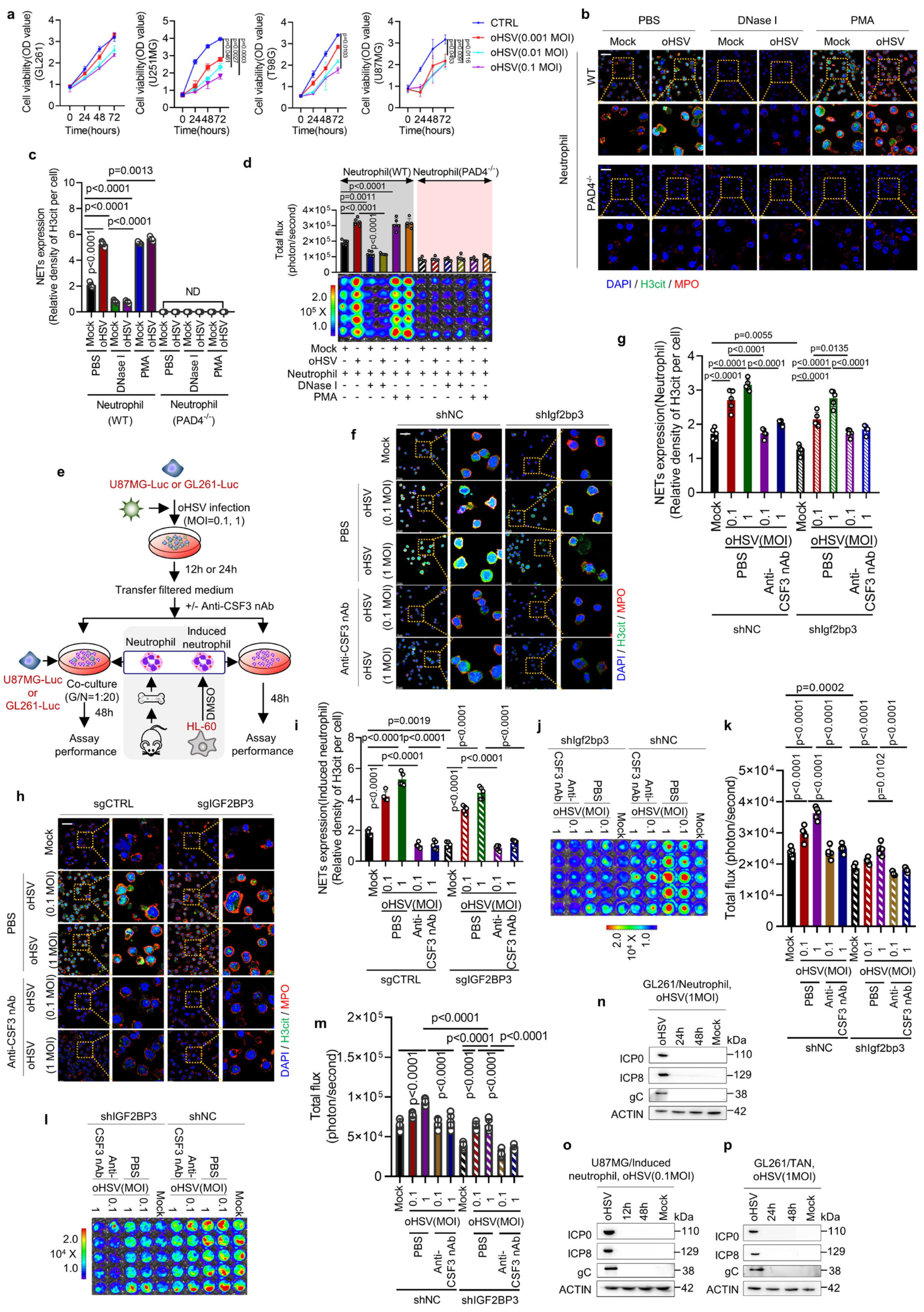

Supplementary Figure 4. oHSV promotes IGF2BP3 induced NETosis and glioma cell survival

(a) Cells were infected with oHSV at the indicated MOI, and cell viability was assessed by CCK8 assay. n = 5 biological independent samples.

(b, c) Representative immunofluorescence images **(b)** and quantification **(c)** of NET formation in the GL261 cell/bone marrow neutrophil uncontacted transwell assay. Scale

bars, 20 μm. Representative images of n = 5. n = 5 biological independent samples.

(d) The NET-induced GL261 cell survival in the contacted co-culture system was measured by luciferase assay. n = 5 biological independent samples.

(e) A schematic representation of the contacted co-culture assay and uncontacted transwell assay is shown. "U87MG-Luc, GL261-Luc" indicates U87MG or GL261 cells expressing luciferase. "Neutrophil" indicates bone marrow neutrophils. "Induced neutrophil" indicates HL-60 cells pretreated with DMSO for 48 hours. "G/N=1:20" indicates the ratio of glioma cells to neutrophils.

(f-i) Representative immunofluorescence images **(f, h)** and corresponding quantifications **(g, i)** of NET formation in the GL261 cell/bone marrow neutrophil co-culture system and the U87MG cell/induced neutrophil co-culture system, respectively. Scale bars, 20 μm. Representative images of n = 5. n = 5 biological independent samples.

(j-m) The NET-induced survival of GL261 cells (j) and U87MG cells **(l)** in the contacted co-culture system, along with corresponding quantifications **(k, m)**, was assessed using luciferase assays. n = 5 biological independent samples.

(n-p) Viral proteins (ICP0, ICP8, and gC) in the supernatant from the three co-culture systems of oHSV-infected tumor cells were analyzed by western blot at the indicated time points. n = 3 biological independent experiments.

Statistical significance was determined using two-way ANOVA in **(a)**, or one-way ANOVA in **(c), (d), (g), (i), (k), (m)**. Data represent the mean ± SD. Source data are provided in the Source Data file.

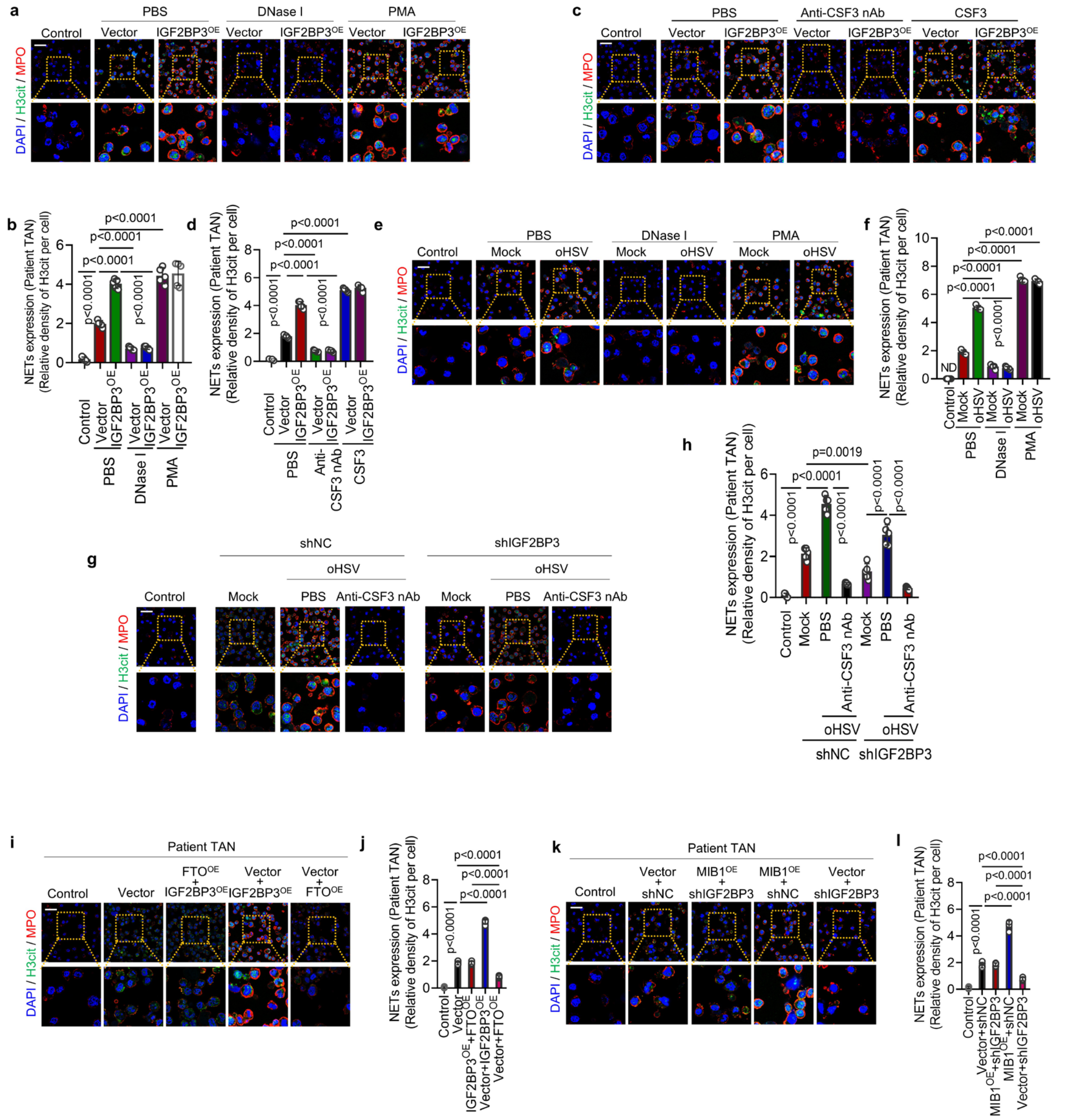

Supplementary Figure 5. oHSV enhances IGF2BP3 induced NETosis in primary cultured glioma cell

(a, b) Representative immunofluorescence images **(a)** and quantification **(b)** of NET formation in the human primary GBM cell/TAN uncontacted transwell assay. "Control" indicates TANs unstimulated with glioma cells in vitro. Scale bars, 20 μm. Representative images of n = 5. n = 5 biological independent samples.

(c, d) Representative immunofluorescence images **(c)** and quantification **(d)** of NET formation in the human primary GBM cell/TAN uncontacted transwell assay treated with CSF3 neutralizing antibody (Anti-CSF3 nAb) or recombinant protein (CSF3). Scale bars are 20 μm. Representative images of n = 5. n = 5 biological independent samples.

(e-l) Representative immunofluorescence images **(e, g, i, k)** and quantification **(f, h, j, l)** of NET formation in the human primary GBM cell/TAN uncontacted transwell assay with the indicated treatments. "ND" as undetected. Scale bars, 20 μm. Representative images of n = 5. n = 5 biological independent samples.

Statistical significance was determined using one-way ANOVA in **(b), (d), (f), (h), (j), (l).**

Data represent the mean ± SD. Source data are provided in the Source Data file.

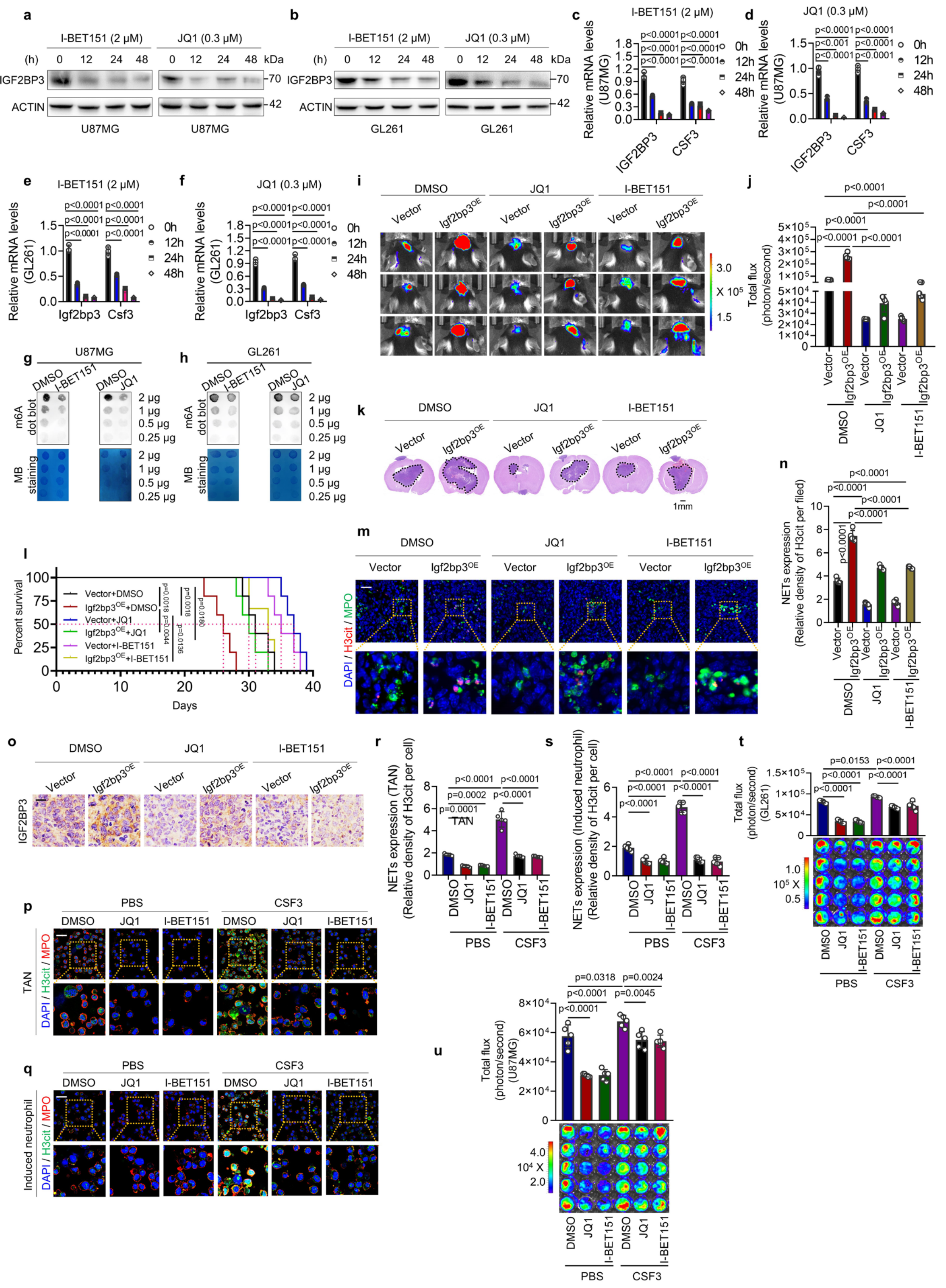

Supplementary Figure 6. Inhibition of IGF2BP3 blocks NETosis and glioma survival

(a, b) The effect of BET inhibitors (JQ1, I-BET151) on IGF2BP3 protein levels in U87MG and GL261 cells was detected by western blot. n = 3 biological independent experiments. **(c-f)** The effect of BET inhibitors (JQ1, I-BET151) on IGF2BP3 and CSF3 mRNA levels in

U87MG and GL261 cells was detected by RT-qPCR. n = 3 biological independent experiments.

(g, h) The effect of BET inhibitors (JQ1, I-BET151) on total m6A levels in U87MG and GL261 cells was measured by m6A dot blot assay. Methylene blue (MB) staining was used as a loading control.

(i) Representative images of luciferase signal in C57BL/6 mice with GL261 tumors after indicated treatments.

(j) Tumor size was estimated by measuring luciferase activity in tumor cells (n = 5 mice per group).

(k) HE staining analysis of tumor volume. The tumor size was indicated with a dashed line.

(l) Kaplan-Meier analysis was performed on a mouse model with intracranially implanted tumors derived from GL261 with indicated treatments to assess survival (n=5 mice per group).

(m, n) Representative immunofluorescence images **(m)** and quantification **(n)** of NET formation in the indicated tumor tissues. Scale bars, 20 μm. Representative images of n = 5. n = 5 biological independent samples.

(o) Immunohistochemistry analysis was conducted to evaluate IGF2BP3 expression in the indicated tumor tissues. Scale bars, 20 μm. Representative images of n = 5.

(p-s) Representative immunofluorescence images **(p, q)** and corresponding quantifications **(r, s)** of NET formation were obtained in the GL261 cell/TAN and the U87MG cell/induced neutrophil (HL-60) uncontacted transwell assay, respectively. Scale bars, 20 μm. Representative images of n = 5. n = 5 biological independent samples.

(t, u) The effect of NETs on GL261 **(t)** and U87MG **(u)** cell survival in contacted co-culture system was detected by luciferase assay. Representative images of n = 5. n = 5 biological independent samples.

Statistical significance was determined using one-way ANOVA in **(c-f), (j), (n), (r-u)**, or the log-rank (Mantel-Cox) test in **(l)**. Data represent the mean ± SD. Source data are provided in the Source Data file.

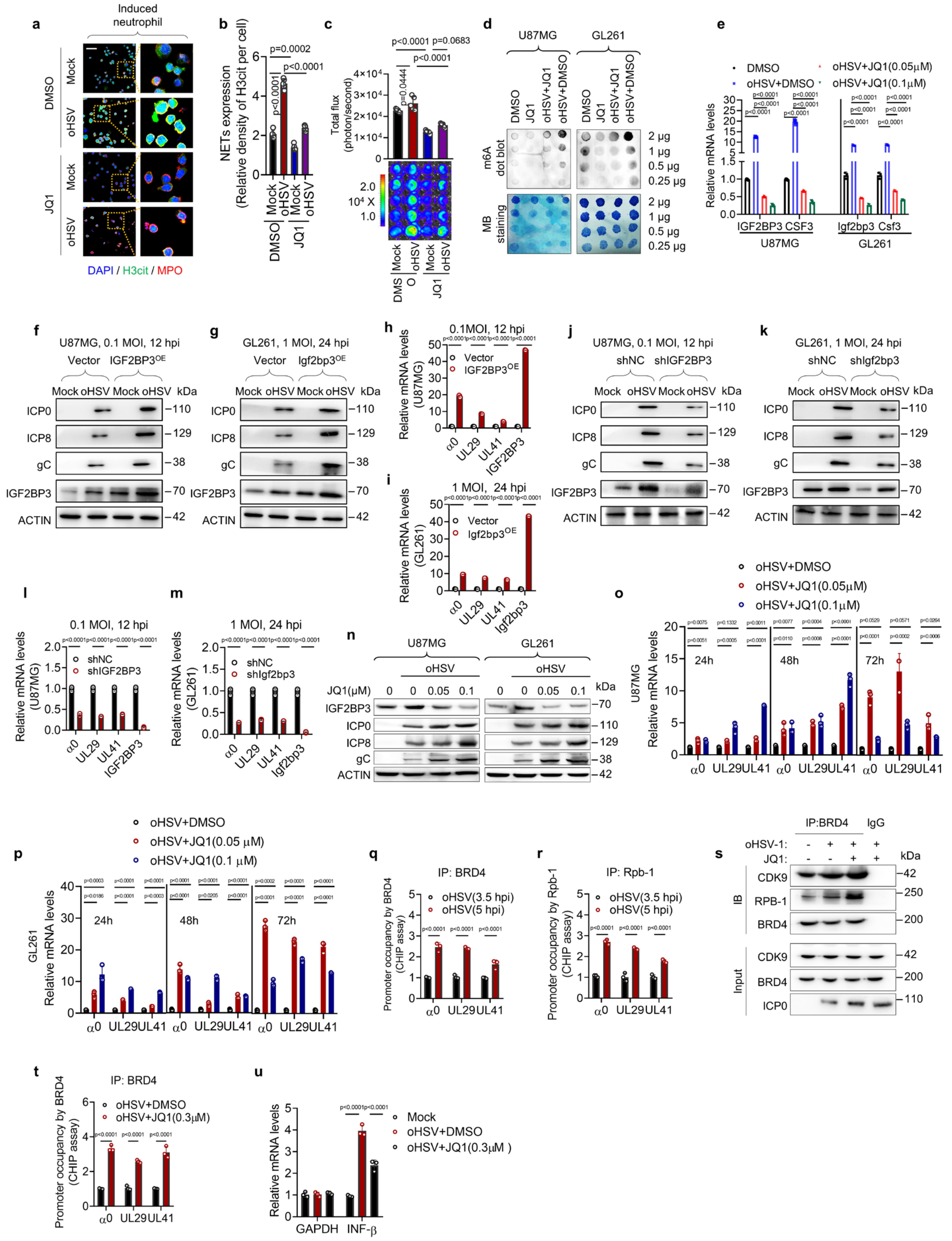

Supplementary Figure 7. BET inhibitors promote oHSV growth and block oHSV-induced NETosis

(a, b) Representative immunofluorescence images **(a)** and quantification **(b)** of NET formation in U87MG cell/induced neutrophil co-culture system. Scale bars, 20 μm. Representative images of n = 5. n = 5 biological independent samples.

(c) The effect of NETs on U87MG cell survival in co-culture system was detected by luciferase assay. n = 5 biological independent samples.

(d) The effect of JQ1 on total m6A levels was detected. Methylene blue (MB) staining was

used as a loading control.
(e) The effect of JQ1 on the mRNA levels of IGF2BP3(Igf2bp3) and CSF3(Csf3) was detected in U87MG and GL261 cells. n = 3 biological independent experiments.
(f, g, j, k) The impact of IGF2BP3 on the protein expression of oHSV viral genes was detected. n = 3 biological independent experiments.
(h, i, l, m) The effect of IGF2BP3 (Igf2bp3) on the mRNA levels of oHSV viral genes was detected. n = 3 biological independent experiments.
(n) The effect of JQ1 on the protein levels of IGF2BP3, ICP0, ICP8, and gC was detected. n = 3 biological independent experiments.
(o, p) The effect of JQ1 on the mRNA levels of α0, UL29, and UL41 was detected. n = 3 biological independent experiments.
(q, r) The oHSV-induced enrichment of BRD4 and RPB-1 recruited to the promoters of oHSV viral genes in U87MG cells was detected. Cells were infected with oHSV at 5 MOI for the indicated times. n = 3 biological independent experiments.
(s) The recruitment of BRD4 to the CDK9/RPB-1 complex in oHSV-infected U87MG cells. n = 3 biological independent experiments.
(t) The recruitment of BRD4 to the promoters of oHSV viral genes in U87MG cells. n = 3 biological independent experiments.
(u) The mRNA levels of GAPDH and IFN-β in U87MG cells. n = 3 biological independent experiments.
For panels s-u, cells were infected with oHSV (5 MOI) and simultaneously treated with either DMSO or JQ1 (0.3 μM) for 5 hours.
Statistical significance was determined using one-way ANOVA in **(b), (c), (e), (o), (p), (u),** or two-tailed Student's t-test in **(h), (i), (l), (m), (q), (r), (t)**. Data represent the mean ± SD. Source data are provided in the Source Data file.

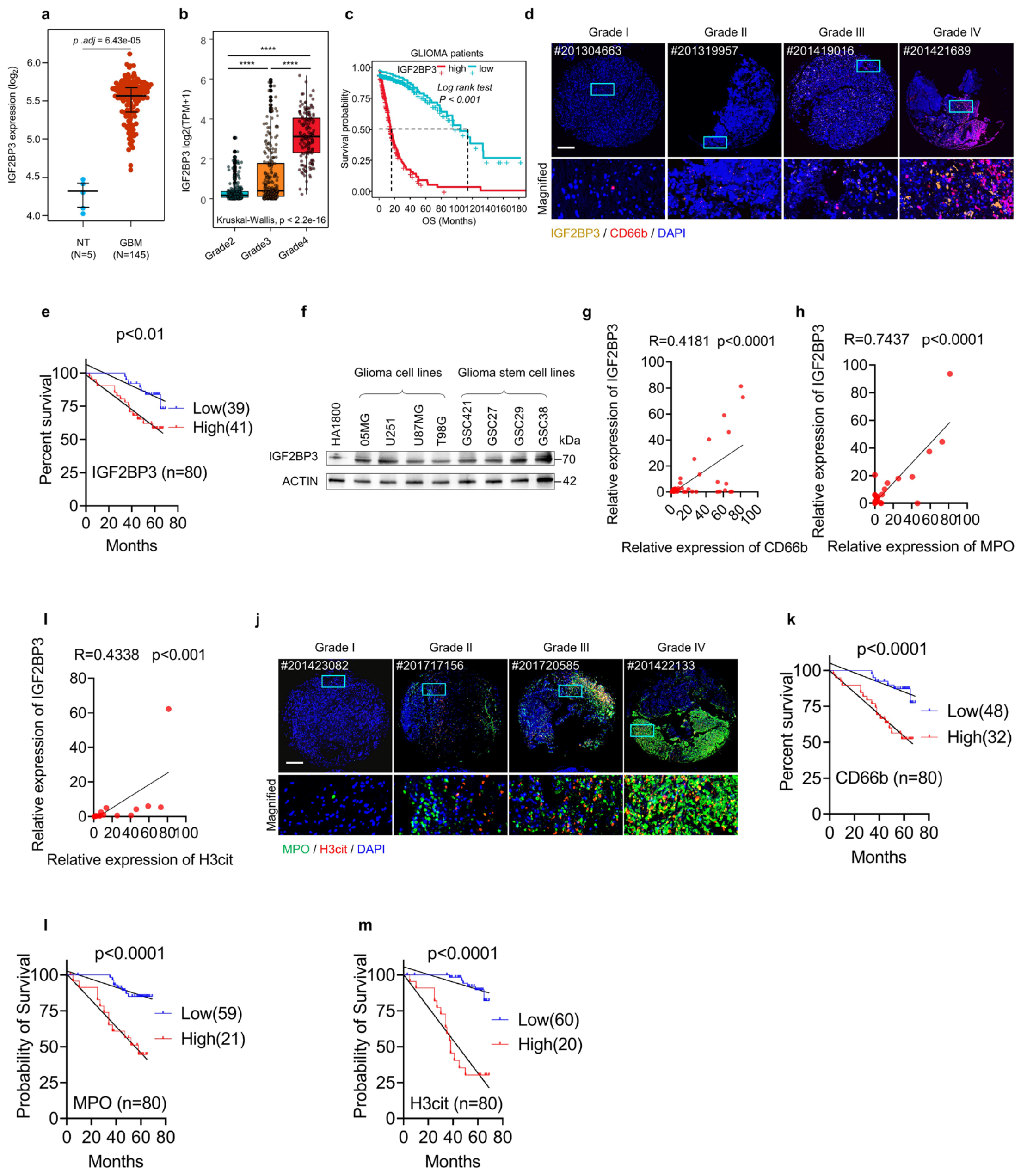

Supplementary Figure 8. IGF2BP3-induced NET is linked to poor prognosis in glioma patients

(a) Bioinformatic analysis of TCGA database revealed the relative mRNA levels of IGF2BP3 in normal brain tissues(n=5) and glioblastoma multiforme (GBM) tumor samples(n=145).

(b) Bioinformatics analysis of TCGA database showed the differential expression of IGF2BP3 among different glioma grades. A total of 596 TCGA glioma patient samples were included in this analysis (Grade 2=211; Grade 3=233; Grade 4=152).

(c) Kaplan-Meier survival analysis of GBM patients based on their IGF2BP3 expression levels. The “surv-cutpoint” function from the “survival” R package was applied to stratify samples into IGF2BP3-High and -Low groups. n = 596 (High=254; Low=342) glioma patients.

(d, j) Representative immunofluorescence images showing the expression of IGF2BP3,

CD66b, MPO, and H3cit in different glioma grades. Scale bars, 20 μm. n = 80 glioma patient samples.

(e, k-m) Kaplan-Meier survival analysis of GBM patients based on their expression levels of IGF2BP3, CD66b, MPO and H3cit, respectively. n = 80 glioma patients.

(f) Western blotting analysis of IGF2BP3 expression in human normal astrocyte cell line HA1800, human glioma cell lines, and glioma stem cell lines. n = 3 biological independent experiments.

(g-i) Spearman's correlation analysis of IGF2BP3 expression with CD66b, MPO, and H3cit expression in glioma patient tissues. n = 80 glioma patients.

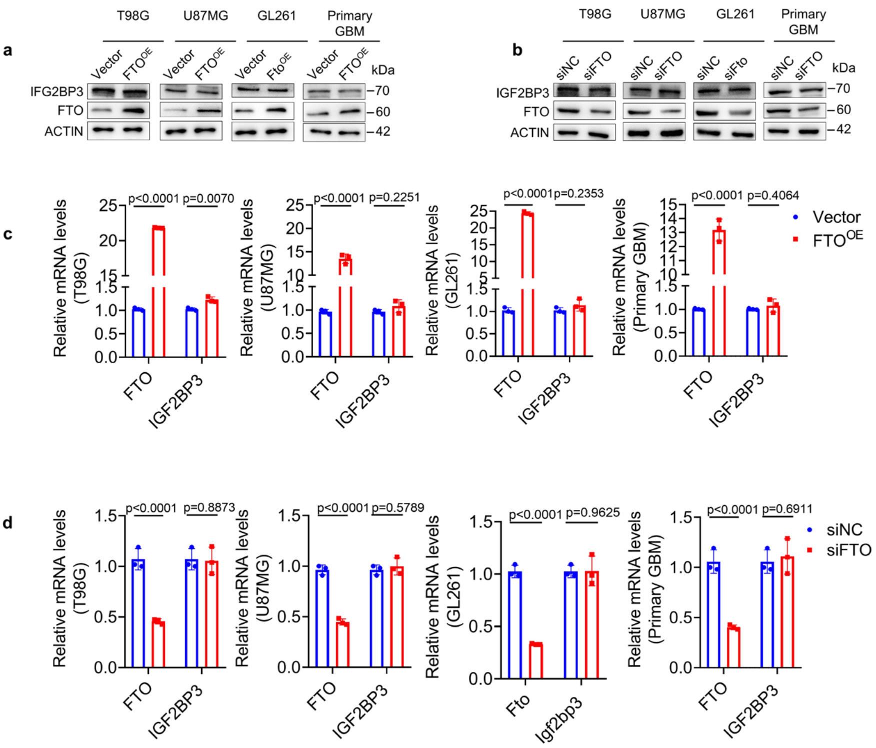

Supplementary Figure 9. FTO does not regulate IGF2BP3 expression in different cell lines

(a, b) The impact of FTO overexpression or knockdown on the IGF2BP3 protein expression in T98G, U87MG, GL261 and human primary GBM cells. n = 3 biological independent experiments.

(c, d) The impact of FTO overexpression or knockdown on the IGF2BP3 mRNA levels in T98G, U87MG, GL261 and human primary GBM cells. n = 3 biological independent experiments.

Statistical significance was determined using two-tailed Student's t-test in **(c), (d).** Data represent the mean ± SD. Source data are provided in the Source Data file.

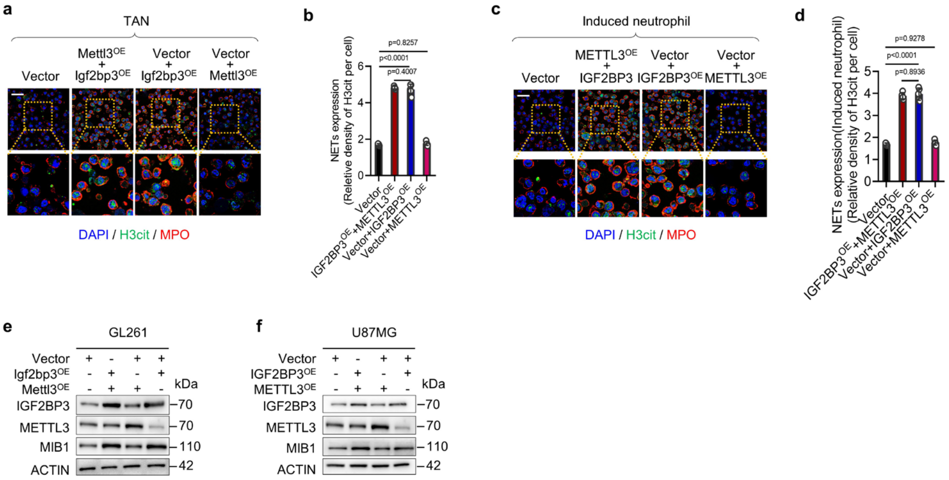

Supplementary Figure 10. METTL3 has little effect on IGF2BP3-mediated NETosis

(a, b) Representative immunofluorescence images **(a)** and quantification **(b)** of NET formation in an uncontacted transwell assay of GL261 cells and TANs with the indicated treatment. Scale bars, 20 μm. Representative images of n = 5. n = 5 biological independent samples.

(c, d) Representative immunofluorescence images **(c)** and quantification **(d)** of NET formation in an uncontacted transwell assay of U87MG cells and induced neutrophils with the indicated treatment. Scale bars, 20 μm. Representative images of n = 5. n = 5 biological independent samples.

(e, f) Rescue experiments showed the effects of METTL3 interruption on IGF2BP3/MIB1 pathway in GL261**(e)** and U87MG**(f)** cells. n = 3 biological independent experiments.

Supplementary Table 1. The sequence of oligonucleotides used in this study.

**qPCR primers**	**Forward primer sequences (5'-3')**	**Reverse primer sequence (5'-3')**
Human *METTL3*	AGGCAGCTCATCTGTGTCCT	GCTTGGCGTGTGGTCTTT
Mouse *Mettl3*	CGTGGAGCTCTATCCAGGCC	GGCAGTAGGCACGGGACTAT
Human *IGF2BP3*	TGCGGCTTGTAAGTCTATTCTG	AGTGTCTGTGTCTTGCTCAATT
Mouse *Igf2bp3*	CCACCAGAGGCTCAGTTCAAGG	AGCAGCAAAGGACGGCACTC
Human *MIB1*	ATGTGCTGTGGAGGGAAAAG	GACACACAGGGCACATTGTC
Mouse *Mib1*	CCTACGACCTGCGTATCCTG	ACCTTTCCTCTACGCCCATT
Human *FTO*	ACTTGGCTCCCTTATCTGACC	TGTGCAGTGTGAGAAAGGCT
Mouse *Fto*	GAGCAGCCTACAACGTGACT	GAAGCTGGACTCGTCCTCAC
Human *CSF3*	GCTGCTTGAGCCAACTCCATA	GAACGCGGTACGACACCTC
Mouse *Csf3*	GCCACCTACAAGCTGTGTCACC	GCTGGCTTAGGCACTGTGTCTG
Human *ACTIN*	ACTGGAACGGTGAAGGTGAC	AGAGAAGTGGGGTGGCTTTT
Mouse *Actin*	GACGGCCAGGTCATCACTATTG	AGGAAGGCTGGAAAAGAGCC
**gRNA/si/shRNA sequences**	**Sequence (5'-3')**	
Human *IGF2BP3* gRNA	GGAAGACTGGTGGATGCGTT	
Human *IGF2BP3* shRNA1	GTCGATTACTATCCTCTCTAC	
Human *IGF2BP3* shRNA2	GCAAAGGATTCGGAAACTTCA	
Mouse *Igf2bp3* shRNA1	CCTCGGACCTAGAAAGTATCT	

Mouse *Igf2bp3* shRNA2	GGAGATTATGCATAAGGAAGC	
Human *FTO* siRNA1	TCACCAAGGAGACTGCTATTT	
Human *FTO* siRNA2	AAAUAGCCGCUGCUUGUGAGA	
Mouse *Fto* siRNA1	CTAGGGTTTTGCTTCCAGAATT	
Mouse *Fto* siRNA2	ACGAAUUGCCCGAACAUUA	
**Chip-qPCR primers**	**Forward primer sequences (5'-3')**	**Reverse primer sequence (5'-3')**
HSV-α0	ATAAGTTAGCCCTGGCCCCGA	GCTGCGTCTCGCTCCG
HSV-*UL29*	CCACGCCCACCGGCTGATGAC	TGCTTACGGTCAGGTGCTCCG
HSV-*UL41*	ATACCATAATTTTATTGGTGGGTCG	CGACCCACCAATAAAATTATGGTAT
Human *GAPDH*	TTCGACAGTCAGCCGCATCTTCTT	CAGGCGCCCAATACGACCAAATC
Human *IFN-β*	TAGTCATTCACTGAAACTTTA	AGGTTGCAGTTAGAATGTC

Supplementary Table 2. Key resources table.

REAGENT or RESOURCE	SOURCE	IDENTIFIER
	**Antibodies**	
Rabbit anti-IGF2BP3 antibody	ABclonal	Cat.: #A4444
Rabbit anti-METTL3 antibody	Proteintech Group	Cat.: #15073-1-AP
Rabbit anti-METTL14 antibody	Proteintech Group	Cat.: #26158-1-AP
Rabbit anti-FTO antibody	Proteintech Group	Cat.: #27226-1-AP
Rabbit anti-ALKBH5 antibody	Proteintech Group	Cat.: #16837-1-AP
Rabbit anti-beta-Actin antibody	Proteintech Group	Cat.: #23660-1-AP
Anti-rabbit IgG	Cell signaling technology	Cat.: # 7054S
Anti-mouse IgG	Cell signaling technology	Cat.: #7056S
Anti-mouse IgG(H+L), F(ab')2 Fragment (Alexa Fluor® 555 Conjugate)	Cell signaling technology	Cat.: ##4409
Anti-rabbitIgG (H+L), F(ab')2 Fragment (Alexa Fluor® 488 Conjugate)	Cell signaling technology	Cat.: # 4412S
FLAG-Tag	Sigma-Aldrich	Cat.: #F2555
HA-Tag	Cell signaling technology	Cat.: #3724S
Rabbit anti-CD66b antibody	Proteintech Group	Cat.: #19496-1-AP
Rabbit anti-MPO antibody	Proteintech Group	Cat.: # 222251-AP
Rabbit anti-CltH3 antibody	abcam	Cat.: #ab281584
Rabbit anti-MIB1 antibody	Proteintech Group	Cat.: #11893-1-AP
Rabbit anti-HUWE1 antibody	Proteintech Group	Cat.: #19430-1-AP
Rabbit anti-HERC2 antibody	Proteintech Group	Cat.: #27459-1-AP
Mouse anti-ICP0 antibody	Provided by Dr. Bernard Roizman（University of Chicago）	
Mouse anti-ICP8 antibody		
Mouse anti-gC antibody		
	**Bacterial and virus strains**	
DH5α Chemically Competent Cell	Tsingke	Cat.: #TSC-C014

Stabl3 Chemically Competent Cell	Tsingke	Cat.: #TSC-C06
BL21 Chemically Competent Cell	Tsingke	Cat.: #TSC-E01
**Chemicals, peptides, and recombinant proteins**		
DPBS	Thermo Fisher Scientifific	Cat# 14040117
Puromycin dihydrochloride	Sigma-Aldrich	Cat# P8833
Actinomycin D	Sigma-Aldrich	Cat# A9415
Cycloheximide	Sigma-Aldrich	Cat# C1988
Recombinant Human CSF3	ABclonal	Cat# RP01722
Recombinant mouse CSF3	ABclonal	Cat# RP00573
Penicillin Streptomycin	Thermo Fisher Scientifific	Cat# 15-140-122
Pierce Protein A/G Magnetic Beads	Thermo Fisher Scientifific	Cat# 88803
Wright-Giemsa Stain	Polysciences	Cat# 24985
Wright-Giemsa Stain/Buffer	Polysciences	Cat# 24984
DMSO	Sigma	Cat#D2650
PrimeSTAR® HS (Premix)	Takara	Cat#R040Q
Trizol	Thermo Fisher Scientifific	Cat#15596018
Fluoromount-G™	YESEN	Cat#36307ES08
Opti-MEM	Gibco	Cat#31985070
T4 DNA ligase	Vazyme	Cat#C301-01
InStab ™ Phosphatase Inhibitor Cocktail	YESEN	Cat#20109ES05
Fetal Bovine Serum	YESEN	Cat#40130ES76
Dulbecco's Modified Eagle Medium	YESEN	Cat#41401ES76
HighGene plus Transfection reagent	ABclonal	Cat#RM09014P
PMA	YESEN	Cat#50601ES03
DNAseI	YESEN	Cat#60613ES70
AceQ qPCR SYBR Green Master Mix	Vazyme	Cat#Q111-02
GoldBand Plus 3-color Regular Range Protein Marker	YESEN	Cat#20350ES72
GoldBand DL2,000 DNA Marker	YESEN	Cat#10501ES60
Agarose	YESEN	Cat#10208ES60
YeaRed Nucleic Acid Gel Stain	YESEN	Cat#10203ES76
Amersham ECL Prime Western Blotting	Thermo Fisher Scientifific	Cat# 45-010-090
MG132	MCE	Cat# HY-13259
JQ1	Selleck	Cat#S7110
IBET-151	Selleck	Cat# S2780
**Critical commercial assays**		
Magna MeRIP m6A Kit	Millipore	Cat# 17-10499-2
Dual Luciferase Reporter Gene Assay Kit	Yesen	Cat# 11402ES80

MojoSort ™ Mouse Neutrophil Isolation Kit	Biolegend	Cat# 480058
Cell Counting Kit-8	Beyotime	Cat# C0040
Mouse G-CSF ELISA Kit	Proteintech Group	Cat# KE10025
Human G-CSF ELISA Kit	Proteintech Group	Cat# KE00106
HiScript III 1st Strand cDNA Synthesis Kit	Vazyme	Cat#R312-01
BCA Protein Quantification Kit	Yesen	Cat#20201ES76
**Experimental models: Cell lines**		
HEK293T	ATCC	CRL-3216
HL60	ATCC	CCL-240
U87MG	ATCC	HTB-14
GL261	ATCC	N/A
T98G	ATCC	CRL-1690
HA1800	ATCC	N/A
U251MG	Provided by Dr. Guangmei Yan（Sun Yat-sen University）	
05MG	Provided by Dr. Guangmei Yan（Sun Yat-sen University）	
GSCs	Provided by Dr. Jianghong Man (National Center of Biomedical Analysis)	
**Experimental models: Organisms/strains**		
C57BL/6 mice	Shanghai Slac Laboratory Animal Co. Ltd	C57BL/6 mice
PAD4-/-mice	Dr. Erwei Song Laboratory	$PAD4^{-/-}$mice
**Recombinant DNA**		
pLVX-IGF2BP3-Flag	This study	N/A
pLVX-igf2bp3-Flag	This study	N/A
pLKO.1-shIGF2BP3	This study	N/A
pLKO.1-shigf2bp3	This study	N/A
pSp-CAS9(BB)-2A-IGF2BP3	This study	N/A
pCDH-FTO-Flag	This study	N/A
pCDH-FTO-N-Flag	This study	N/A
pCDH-FTO-C-Flag	This study	N/A
pCDH-MIB1-HA	This study	N/A
pCDH-MIB1-N-HA	This study	N/A
pCDH-MIB1-C-HA	This study	N/A
pCDH-fto-Flag	This study	N/A
pCDH-fto-N-Flag	This study	N/A
pCDH-fto-C-Flag	This study	N/A
pCDH-mib1-HA	This study	N/A
pCDH-mib1-N-HA	This study	N/A

pCDH-mib1-C-HA	This study	N/A
pGL3-MIB1-WT	This study	N/A
pGL3-MIB1-MUT	This study	N/A
psiCHECK2-CSF3-WT	This study	N/A
psiCHECK2-CSF3-MUT	This study	N/A
pRK5-HA-Ubiqutin-K0		addgene#17603
pRK5-HA-Ubiqutin-K48R		addgene#17604
pRK5-HA-Ubiqutin-K48		addgene#17605
pRK5-HA-Ubiqutin-K63		addgene#17606
pRK5-HA-Ubiqutin-K33		addgene#17607
pRK5-HA-Ubiqutin-wt		addgene#17608
pRK5-HA-Ubiqutin-K6		addgene#22900
pRK5-HA-Ubiqutin-K11		addgene#22901
pRK5-HA-Ubiqutin-K27		addgene#22902
**Software and algorithms**		
ImageJ	NIH	https://imagej.nih.gov/ij/
R Statistical Software version 4.0.5	The R Foundation	https://www.r-project.org/
GraphPad Prism 9	Prism	https://www.graphpad-prism.cn/

References

1. Makowske, M., Ballester, R., Cayre, Y. & Rosen, O. M. Immunochemical evidence that three protein kinase C isozymes increase in abundance during HL-60 differentiation induced by dimethyl sulfoxide and retinoic acid. *J Biol Chem* **263**, 3402-3410, (1988)
