# Supplementary material for: Overcoming therapeutic resistance in oncolytic herpes virotherapy by targeting IGF2BP3-induced NETosis in malignant glioma
Source: Nat Commun. 2024 Jan 2;15:131. doi: 10.1038/s41467-023-44576-2 (PMC10762148; doi:10.1038/s41467-023-44576-2)
Supplement: Supplementary file 3 — Reporting Summary [file 41467_2023_44576_MOESM3_ESM.pdf]

nature portfolio

Corresponding author(s): Minfeng Shu

Last updated by author(s): Nov 27, 2023

Reporting Summary

Nature Portfolio wishes to improve the reproducibility of the work that we publish. This form provides structure for consistency and transparency in reporting. For further information on Nature Portfolio policies, see our Editorial Policies and the Editorial Policy Checklist.

Statistics

For all statistical analyses, confirm that the following items are present in the figure legend, table legend, main text, or Methods section.

n/a	Confirmed	
☐	☒	The exact sample size (*n*) for each experimental group/condition, given as a discrete number and unit of measurement
☐	☒	A statement on whether measurements were taken from distinct samples or whether the same sample was measured repeatedly
☐	☒	The statistical test(s) used AND whether they are one- or two-sided<br>*Only common tests should be described solely by name; describe more complex techniques in the Methods section.*
☐	☒	A description of all covariates tested
☐	☒	A description of any assumptions or corrections, such as tests of normality and adjustment for multiple comparisons
☐	☒	A full description of the statistical parameters including central tendency (e.g. means) or other basic estimates (e.g. regression coefficient) AND variation (e.g. standard deviation) or associated estimates of uncertainty (e.g. confidence intervals)
☐	☒	For null hypothesis testing, the test statistic (e.g. *F*, *t*, *r*) with confidence intervals, effect sizes, degrees of freedom and *P* value noted<br>*Give P values as exact values whenever suitable.*
☒	☐	For Bayesian analysis, information on the choice of priors and Markov chain Monte Carlo settings
☒	☐	For hierarchical and complex designs, identification of the appropriate level for tests and full reporting of outcomes
☒	☐	Estimates of effect sizes (e.g. Cohen's *d*, Pearson's *r*), indicating how they were calculated

Our web collection on statistics for biologists contains articles on many of the points above.

Software and code

Policy information about availability of computer code

Data collection	The RNA sequencing data in transcripts per kilobase million (TPM) format for TCGA-LGG (Lower Grade Glioma; WHO grade II–III) and TCGA-GBM (glioblastoma multiforme; WHO grade IV) were uniformly processed by TOIL and downloaded from UCSC Xena (https://xenabrowser.net/datapage/). Real-time PCR data were collected by Bio-Rad CFX96 Real-Time PCR Detection System and associated software; LC-MS data were collected with Analyst® 1.6.3 Software for QTRAP 6500 LC-MS system (SCIEX); Western blot data were collected by Tanon 4600 Chemiluminescence Imaging System and associated software; Dual-luciferase activity data were collected by the GloMax Luminometer and associated software; Additional information about software was described in the manuscript or available upon request.
Data analysis	Statistical analyses were performed using Graphpad Prism 9; The clinical data from public databases were analyzed using R Statistical Software version 4.0.5, Western blot data and IHC staining data were quantified using Image J.

For manuscripts utilizing custom algorithms or software that are central to the research but not yet described in published literature, software must be made available to editors and reviewers. We strongly encourage code deposition in a community repository (e.g. GitHub). See the Nature Portfolio guidelines for submitting code & software for further information.

Data

Policy information about availability of data

All manuscripts must include a data availability statement. This statement should provide the following information, where applicable:

- Accession codes, unique identifiers, or web links for publicly available datasets
- A description of any restrictions on data availability
- For clinical datasets or third party data, please ensure that the statement adheres to our policy

1. All sequencing data generated in this study have been deposited in GEO repository under accessions GSE235828 (IGF2BP3-seq) https://www.ncbi.nlm.nih.gov/geo/query/acc.cgi?acc=GSM7510198 and GSE235568(U87-HSV-Seq). https://www.ncbi.nlm.nih.gov/geo/query/acc.cgi?acc=GSM7505427.
2.The mass spectrometry proteomics data have been deposited to the ProteomeXchange Consortium (http:// proteomecentral.proteomexchange.org) via the iProX partner repository with the dataset identifier PXD043362. https://proteomecentral.proteomexchange.org/cgi/GetDataset?ID=PXD043362. All the summary or representative data generated and supporting the findings of this study are available within the paper.

Research involving human participants, their data, or biological material

Policy information about studies with human participants or human data. See also policy information about sex, gender (identity/presentation), and sexual orientation and race, ethnicity and racism.

Reporting on sex and gender	The study did not account for gender.
Reporting on race, ethnicity, or other socially relevant groupings	N/A
Population characteristics	Immunohistochemistry on primary glioma samples was performed on 80 patient samples (33 females and 47 males), ages range from 18 to 76 years, and primary GBM cell lines were obtained from 1 male patient sample(age 64).
Recruitment	Patients diagnosed with glioma were recruited. Informed consents were obtained from involved patients. All human specimens were evaluated by two independent pathologists. No self-selection bias or other biases in recruitment.
Ethics oversight	The study related to human research was approved by Human Research Ethics Committee of Zhongshan hospital, Fudan University.

Note that full information on the approval of the study protocol must also be provided in the manuscript.

Field-specific reporting

Please select the one below that is the best fit for your research. If you are not sure, read the appropriate sections before making your selection.

[x] Life sciences [ ] Behavioural & social sciences [ ] Ecological, evolutionary & environmental sciences

For a reference copy of the document with all sections, see nature.com/documents/nr-reporting-summary-flat.pdf

Life sciences study design

All studies must disclose on these points even when the disclosure is negative.

Sample size	No statistical method was used to predetermine the sample size. The sample size was based on similar studies of the research field and the number should meet the criteria for statistical results. For the RNA sequencing data, all treatments were analysed in triplicate. TCGA or public clinical trial data was processed as provided by the public repositories. Sample sizes are references in the corresponding figure and/or legends.
Data exclusions	There were no deliberate data exclusions.
Replication	Reproducibility of experimental findings (wherever applicable) was verified by either considering analyses of multiple patient samples, three types of glioma cell line(U87MG, T98G, GL261), and/or equal to at least or more than 3 biologically independent experiments. All attempts at replication were successful.
Randomization	Mice were age and sex-matched and randomized where appropriate (e.g. prior to initiating treatment for matched conditions). For treatments, tumors were measured and fairly distributed into groups based on size so that each group had the same approx mean growth before experimental perturbation.
Blinding	The tumor measurement was performed with cage labels blinded for treatments. The treatments were performed after the tumor measurement. For in vitro experiments, experiments execution and analysis was performed by different people in the lab.

Reporting for specific materials, systems and methods

We require information from authors about some types of materials, experimental systems and methods used in many studies. Here, indicate whether each material, system or method listed is relevant to your study. If you are not sure if a list item applies to your research, read the appropriate section before selecting a response.

Materials & experimental systems

n/a	Involved in the study	
	☒	Antibodies
	☒	Eukaryotic cell lines
☒		Palaeontology and archaeology
	☒	Animals and other organisms
☒		Clinical data
☒		Dual use research of concern
☒		Plants

Methods

n/a	Involved in the study	
☒		ChIP-seq
☒		Flow cytometry
☒		MRI-based neuroimaging

Antibodies

Antibodies used

Rabbit anti-IGF2BP3 antibody ABclonal Cat.: #A4444, diluted 1:1000
Rabbit anti-METTL3 antibody Proteintech Group Cat.: #15073-1-AP, diluted 1:1000
Rabbit anti-METTL14 antibody Proteintech Group Cat.: #26158-1-AP, diluted 1:1000
Rabbit anti-FTO antibody Proteintech Group Cat.: #27226-1-AP, diluted 1:1000
Rabbit anti-ALKBH5 antibody Proteintech Group Cat.: #16837-1-AP, diluted 1:1000
Rabbit anti-beta-Actin antibody Proteintech Group Cat.: #23660-1-AP, diluted 1:1000
Anti-rabbit IgG Cell signaling technology Cat.: # 7054S, diluted 1:1000
Anti-mouse IgG Cell signaling technology Cat.: #7056S, diluted 1:1000
"Anti-mouse IgG(H+L), F(ab')2Fragment (Alexa Fluor® 555 Conjugate)"Cell signaling technology, Cat.:##4409, diluted 1:200
"Anti-rabbit IgG (H+L), F(ab')2Fragment (Alexa Fluor® 488 Conjugate)" Cell signaling technology,Cat.: #4412S, diluted 1:200
FLAG-Tag Sigma-Aldrich Cat.: #F2555, diluted 1:1000
HA-Tag Cell signaling technology Cat.: #3724S, diluted 1:1000
Rabbit anti-CD66b antibody Proteintech Group Cat.: #19496-1-AP, diluted 1:1000
Rabbit anti-MPO antibody Proteintech Group Cat.: # 222251-AP, diluted 1:1000
Rabbit anti-CltH3 antibody abcam Cat.: #ab281584, diluted 1:1000
Rabbit anti-MIB1 antibody Proteintech Group Cat.: #11893-1-AP, diluted 1:1000
Rabbit anti-HUWE1 antibody Proteintech Group Cat.: #19430-1-AP, diluted 1:1000
Rabbit anti-HERC2 antibody Proteintech Group Cat.: #27459-1-AP, diluted 1:1000
Mouse anti-ICP0 antibody lab-made, diluted 1:1000
Mouse anti-ICP8 antibody lab-made, diluted 1:1000
Mouse anti-gC antibody lab-made, diluted 1:1000
Rabbit anti-BRD4 antibody Abclonal Cat.: #A12677, diluted 1:1000
Mouse anti-CDK9 antibody Santa Cruz Cat.: #sc-13130, diluted 1:200
Anti-rabbit Rpb-1 Cell signaling technology Cat.: # 2629, diluted 1:1000

Validation

anti-IGF2BP3 antibody ABclonal Cat.: #A4444 Species: Rabbit; Application:WB ;
Manufacturer's website: https://abclonal.com.cn/catalog/A4444
anti-METTL3 antibody Proteintech Group Cat.: #15073-1-AP Species: Rabbit; Application:WB ;
Manufacturer's website: https://www.ptgcn.com/products/METTL3-Antibody-15073-1-AP.htm
anti-METTL14 antibody Proteintech Group Cat.: #26158-1-AP Species: Rabbit; Application:WB ;
Manufacturer's website: https://www.ptgcn.com/products/METTL14-Antibody-26158-1-AP.htm
anti-FTO antibody Proteintech Group Cat.: #27226-1-AP Species: Rabbit; Application:WB ; IP
Manufacturer's website: https://www.ptgcn.com/products/FTO-Antibody-27226-1-AP.htm
anti-ALKBH5 antibody Proteintech Group Cat.: #16837-1-AP Species: Rabbit; Application:WB ;Manufacturer's website:
https://www.ptgcn.com/products/ALKBH5-Antibody-16837-1-AP.htm
anti-beta-Actin antibody Proteintech Group Cat.: #23660-1-AP Species: Rabbit; Application:WB ; Manufacturer's website:
https://www.ptgcn.com/products/ACTA1-Antibody-23660-1-AP.htm
Anti-rabbit IgG Cell signaling technology Cat.: # 7054S Species: Rabbit; Application:WB ;Manufacturer's website:
https://www.cellsignal.cn/products/secondary-antibodies/anti-rabbit-igg-ap-linked-antibody/7054
Anti-mouse IgG Cell signaling technology Cat.: #7056S Species:Mouse ; Application:WB ;Manufacturer's website:
https://www.cellsignal.cn/products/secondary-antibodies/anti-mouse-igg-ap-linked-antibody/7056
"Anti-mouse IgG(H+L), F(ab')2Fragment (Alexa Fluor® 555 Conjugate)"Cell signaling technology Cat.:##4409 Species: Mouse ; Application:IF ;Manufacturer's website:
https://www.cellsignal.cn/products/secondary-antibodies/anti-mouse-igg-h-l-f-ab-2-fragment-alexa-fluor-555-conjugate/4409
"Anti-rabbitIgG (H+L), F(ab')2Fragment (Alexa Fluor® 488 Conjugate)" Cell signaling technology Cat.: #4412S Species: Rabbit; Application:IF ; Manufacturer's website:
https://www.cellsignal.cn/products/secondary-antibodies/anti-rabbit-igg-h-l-f-ab-2-fragment-alexa-fluor-488-

conjugate/4412FLAG-Tag Sigma-Aldrich Cat.: #F2555 Species: Rabbit; Application:WB;IP ; Manufacturer's website:
https://www.sigmaaldrich.cn/CN/zh/search/f2555
HA-Tag Cell signaling technology Cat.: #3724S Species: Rabbit; Application:WB;IP ; Manufacturer's website:
https://www.cellsignal.cn/products/primary-antibodies/ha-tag-c29f4-rabbit-mab/3724
anti-CD66b antibody Proteintech Group Cat.: #19496-1-AP Species: Rabbit; Application:IF;IHC ; Manufacturer's website:
https://www.ptgcn.com/products/CEACAM3-Specific-Antibody-19496-1-AP.htm
anti-MPO antibody Proteintech Group Cat.: # 222251-AP Species: Rabbit; Application:IF;IHC ; Manufacturer's website:
https://www.ptgcn.com/products/CASP1-Antibody-22915-1-AP.htm
anti-CItH3 antibody abcam Cat.: #ab281584 Species: Rabbit; Application:IF; Manufacturer's website:
https://www.abcam.cn/products/primary-antibodies/histone-h3-citrulline-r2--r8--r17-antibody-rm1001-ab281584.html
anti-MIB1 antibody Proteintech Group Cat.: #11893-1-AP Species: Rabbit; Application:WB;IP; Manufacturer's website:
https://www.ptgcn.com/products/MIB1-Antibody-11893-1-AP.htm
Rabbit anti-HUWE1 antibody Proteintech Group Cat.: #19430-1-AP Species: Rabbit; Application:WB;IP; Manufacturer's website:
https://www.ptgcn.com/products/HUWE1-Antibody-19430-1-AP.htm
anti-HERC2 antibody Proteintech Group Cat.: #27459-1-AP Species: Rabbit; Application:WB;IP; Manufacturer's website:
https://www.ptgcn.com/products/HERC2-Antibody-27459-1-AP.htm
Mouse anti-ICP0 antibody,Mouse anti-ICP8 antibody ,Mouse anti-gC antibody were generously gifted by Dr. Bernard Roizman (University of Chicago)
Rabbit anti-BRD4 antibody Abclonal Cat.: #A12677 Species: Rabbit; Application:WB;IP;
Manufacturer's website:https://abclonal.com.cn/catalog/A12677
Mouse anti-CDK9 antibody Santa Cruz Cat.: #sc-13130 Species: Mouse; Application:WB;IP;
Manufacturer's website:https://www.scbt.com/p/cdk9-antibody-d-7?requestFrom=search
Anti-rabbit Rpb-1 Cell signaling technology Cat.: # 2629 Species: Rabbit; Application:WB;IP;
Manufacturer's website:https://www.cellsignal.com/products/primary-antibodies/rpb1-ctd-4h8-mouse-mab/2629

Eukaryotic cell lines

Policy information about cell lines and Sex and Gender in Research

Cell line source(s)	HEK293T, HL60, HA1800, U87MG, GL261, and T98G cells were obtained from the American type culture collection (ATCC). U251MG and 05MG glioma cells were gifted from Dr. Guangmei Yan (Sun Yat-sen University). Primary GBM patient-derived cells were obtained from one male glioma patient. Patient-derived glioblastoma stem cells (GSCs) used in supplementary figure 8 were generously gifted from Dr. Jianghong Man (National Center of Biomedical Analysis).
Authentication	Cell lines were authenticated by company that we ordered from. Morphology check by microscope and growth curve analysis were performed periodically. Cell morphology is similar to published pictures and cells less than 15 passages were used in study.
Mycoplasma contamination	Mycoplasma free under tested condition.
Commonly misidentified lines (See ICLAC register)	No commonly misidentified cell lines.

Animals and other research organisms

Policy information about studies involving animals; ARRIVE guidelines recommended for reporting animal research, and Sex and Gender in Research

Laboratory animals	Female PAD4-/- mice at the age of six to eight weeks were generously gifted by Dr. Erwei Song (Sun Yat-sen University). Female C57BL/6 mice aged six to eight weeks, were purchased from Shanghai Slac Laboratory Animal Co. Ltd. Animals were maintained in pathogen-free environments at 20–21°C with 60–70% relative humidity on a 12-h light/12-h dark cycle.
Wild animals	No wild animals were used in this study.
Reporting on sex	No
Field-collected samples	No field collected samples were used in the study.
Ethics oversight	The procedures related to animal subjects were approved by the Institute of Animal Care and Use Committee of Fudan University (IACUC: no. 20220228-089; 20230301-071).

Note that full information on the approval of the study protocol must also be provided in the manuscript.

Plants

Seed stocks	*Report on the source of all seed stocks or other plant material used. If applicable, state the seed stock centre and catalogue number. If plant specimens were collected from the field, describe the collection location, date and sampling procedures.*
Novel plant genotypes	*Describe the methods by which all novel plant genotypes were produced. This includes those generated by transgenic approaches, gene editing, chemical/radiation-based mutagenesis and hybridization. For transgenic lines, describe the transformation method, the number of independent lines analyzed and the generation upon which experiments were performed. For gene-edited lines, describe the editor used, the endogenous sequence targeted for editing, the targeting guide RNA sequence (if applicable) and how the editor was applied.*
Authentication	*Describe any authentication procedures for each seed stock used or novel genotype generated. Describe any experiments used to assess the effect of a mutation and, where applicable, how potential secondary effects (e.g. second site T-DNA insertions, mosiacism, off-target gene editing) were examined.*
